# Segmentation of Pulmonary Vascular Trees from Thoracic 3D CT Images

**DOI:** 10.1155/2009/636240

**Published:** 2009-12-14

**Authors:** Hidenori Shikata, Geoffrey McLennan, Eric A. Hoffman, Milan Sonka

**Affiliations:** Iowa Institute for Biomedical Imaging, The University of Iowa, Iowa City, IA 52242, USA

## Abstract

This paper describes an algorithm for extracting pulmonary vascular trees (arteries plus veins) from three-dimensional (3D) thoracic computed tomographic (CT) images. The algorithm integrates tube enhancement filter and traversal approaches which are based on eigenvalues and eigenvectors of a Hessian matrix to extract thin peripheral segments as well as thick vessels close to the lung hilum. The resultant algorithm was applied to a simulation data set and 44 scans from 22 human subjects imaged via multidetector-row CT (MDCT) during breath holds at 85% and 20% of their vital capacity. A quantitative validation was performed with more than 1000 manually identified points selected from inside the vessel segments to assess true positives (TPs) and 1000 points randomly placed outside of the vessels to evaluate false positives (FPs) in each case. On average, for both the high and low volume lung images, 99% of the points was properly marked as vessel and 1% of the points were assessed as FPs. Our hybrid segmentation algorithm provides a highly reliable method of segmenting the combined pulmonary venous and arterial trees which in turn will serve as a critical starting point for further quantitative analysis tasks and aid in our overall goal of establishing a normative atlas of the human lung.

## 1. Introduction

The pulmonary arterial and venous structures deliver deoxygenated blood to the lung periphery and return oxygenated blood to the systemic circulation. These highly complex branching structures support the primary function of the lung that is to bring blood into close proximity with incoming fresh gas delivered to the terminal air sacs (alveoli) through the process of respiration. In clinical practice, it is of great importance, for instance, to be able to characterize the vascular trees for the detection of pulmonary emboli (localized blockages), detection of signs of pulmonary hypertension, and for the differentiation between vasculature and focal opacities (for detection of lung cancer and other localized pathologies). The vascular trees can also serve as a roadmap for the tracking of lung tissues across lung volume changes or across time as the lung is serially monitored. Recent advances in MDCT scanner technology enable the scanning of the entire lung with nearly isotropic submillimeter voxel dimensions (on the order of 0.4 mm). Vessel segments with radii of 2 mm or less are readily detectible in those images. While detectible, manual segmentation of these complex tree structures, even if an individual were willing to take the time, has been found to present a near impossible task due to the following reasons.

- It is difficult to determine boundaries of a vessel consistently, especially thin segments, due to the partial volume effects and image noise.
- Volumetric lung scans of the adult human consist of more than 500 slices and the vascular tree, in a bipodial fashion, rapidly branch as one tracks the vessels from their central to peripheral locations, with the full tree structure consisting of more than 23 generations.

In addition, manual measurements of the vessels for assessment of diameters and branching angles are unreliable. The measurement of diameter requires the determination of cross-sectional planes perpendicular to the local segment centerlines. Similarly, a plane that includes both parent and child segments around the branchpoint needs to be localized for branching angle measurements. Both measurements are difficult to perform manually via 2D images because of problems of for-shortening in projected view. Therefore, highly automated segmentation of the pulmonary vascular tree based on 3D image analysis plays an important role in detecting and characterizing the vessel structure. The segmentation results are sought as we seek to build a lung atlas [1] in which we will establish normative values against which an individual can be compared for the detection of disease. 

 There is great interest in identifying branchpoints of the vascular trees as a set of landmarks that may allow matching of the lung across volume changes [1]. During a respiratory cycle, the lung nonrigidly deforms its shape and individual lobes rotate independent of each other [2]. Branchpoints of the vascular tree can serve as possible landmarks inside the lung. Even though the registration may require separation of arterial and venous trees, segmentation of the entire vascular trees provides branchpoint information required for this purpose. Eventually the arterial and venous trees can readily be separated by scanning during the infusion of iodinated contrast agent and scanning during the arterial phase of the infusion. 

 Several 3D vessel segmentation algorithms have been presented to date. Tube enhancement filters based on a combination of the eigenvalues of a Hessian matrix have been reported in [3–5]. Segmentation can be performed simply by thresholding of the filter output. The filters have an ability to handle a range of radii by multiscale implementation. Lorigo et al. [6] reported a vessel segmentation algorithm based on a “codimension two” level set method. Vasilevskiy and Siddiqi [7] used gradient flows implemented using a level set method for 2D and 3D vessel segmentation. Aylward and Bullitt [8] reported an intensity ridge traversal method to extract vessels. A tracking direction was estimated by an eigenvector of the Hessian matrix at each tracking front position. Boldak et al. reported model-based vessel tracking [9]. Mayer et al. presented pulmonary vessel segmentation in contrast-enhanced CT data [10]. Fridman et al. [11] used cores [12, 13] to track the vascular tree from a seed point. Agam et al. reported a method for vascular tree segmentation using correlation-based enhancement filters and a fuzzy shape representation of the data [14]. An approach based on mathematical morphology and discrete geometry operators was recently reported in [15]. Use of vascular tree segmentation for detection, segmentation, and analysis of pulmonary lobes and sublobes was presented in [16]. 

 The eigenvalues and eigenvectors of the Hessian matrix are implicitly or explicitly used in some of the above algorithms and the algorithms have worked well for extracting vessels in several organ systems imaged by CT or MR. The tube enhancement filters can extract both thin segments and thick segments without using seed points. However, such filters produce disconnections around the junctions since they are based on a cylindrical vessel segment model. Segmentation results obtained from vessel traversal algorithms generally have better connectivity between segments but often miss peripheral thin segments. Our goal outlined in this paper has been to develop an algorithm that extracts peripheral thin segments as well as thick segments from thoracic CT images with better connectivity. The major contribution of the reported work is the development of an algorithm which extracts detailed pulmonary vascular trees by a novel integration of the tube enhancement filter and vessel traversal approaches. Our approach builds on several previously developed and proven methods for vessel enhancement and vessel traversal. However, when applying these algorithms individually to extract pulmonary vessels, several additional issues need to be resolved, which is the topic of this paper. The presented method integrates existing conceptual modules in a way that the final approach is free of the inherent limitations of the individual building blocks.

## 2. Method

Our integrated algorithm consists of three major steps: (1) tube enhancement based on the cylindrical shape model using an eigenvalue of the Hessian matrix serves as a filter to extract vessels and to produce information that is used to determine a set of seed points in the following vessel traversal step. (2) The traversal step starts from each seed point until one of the eigenvalues of the Hessian matrix changes its sign twice, signifying that the front point of a trajectory has reached a junction. (3) Branchpoint analysis is accomplished by applying a thinning method which then allows for the selection of objects with many branchpoints, serving as a means of distinguishing between vascular trees and noise components.

### 2.1. Vessel Enhancement

A vessel segment in a 3D image is often modeled as having a cylindrical shape with a 2D Gaussian-like intensity distribution within its cross-sectional plane. A combination of the eigenvalues of the Hessian matrix is often used to characterize and enhance its shape in the image. Since pulmonary vessels consist of segments with a wide range of radii, a multiscale approach needs to be considered. In this vessel enhancement process, important components to be discussed include

- the filter output function.
- segment radius information for multiscale integration.

#### 2.1.1. Hessian Matrix-Based Vessel Enhancement

**Figure 1:**
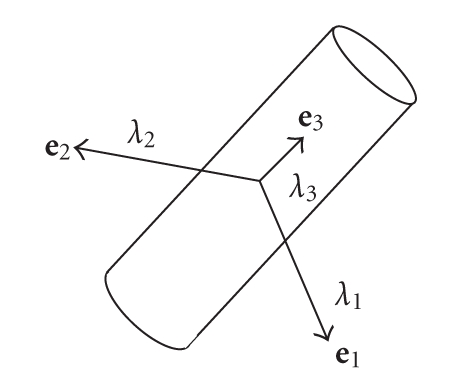
An illustration of a vessel model. It is assumed that the model has a cylindrical shape with a 2D Gaussian-like intensity distribution at each cross section. The arrows show the eigenvectors of a Hessian matrix at the center. The length of the arrows represents the absolute value of corresponding eigenvalues.

Based on the cylindrical vessel model, the eigenvalues of the Hessian matrix are commonly employed recently as efficient criteria to differentiate tube structures from other image components. Let three eigenvalues of the Hessian matrix at a point be *λ*_1_, *λ*_2_, and *λ*_3_ and let their corresponding eigenvectors be **e**_1_, **e**_2_, and **e**_3_, respectively. Suppose that the eigenvalues meet the condition *λ*_1_ ≤ *λ*_2_ ≤ *λ*_3_. Based on the model, when a point is close to the center of a segment, *λ*_1_ and *λ*_2_ take on large negative values whereas *λ*_3_ takes on a small value. Figure 1 illustrates a cylindrical vessel model, with the eigenvalues/eigenvectors at the center of the model. According to the model, the following criteria are typically used to construct a filter output function.

1. Locality: *λ*_1_ and *λ*_2_ are large negative values.
2. Elongation: *λ*_3_ is much less than *λ*_1_ and *λ*_2_ (*λ*_3_/*λ*_1_ ≈ 0 or *λ*_3_/*λ*_2_ ≈ 0).
3. Symmetry: *λ*_1_ and *λ*_2_ are of almost the same values when a point is close enough to the center of a segment (*λ*_2_/*λ*_1_ ≈ 1).

**Figure 2:**
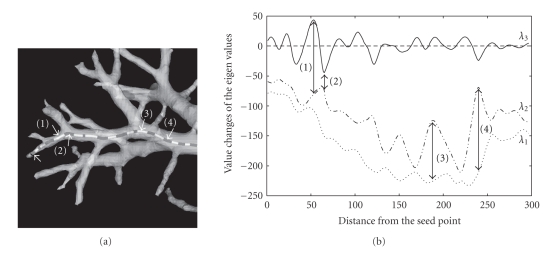
A trajectory of vessel segments and value changes of the eigenvalues along the trajectory. Left-most white arrow in (a) shows the starting point of the trajectory that advances left to right. The trajectory is calculated with fixed *σ*_*f*_ = 1. *λ*_3_ oscillates as the front advances and takes negative values close to the junctions indicated by dark gray regions on the trajectory. *λ*_3_ takes large values at location (1) and (2) making absolute value of the ratio *λ*_3_/*λ*_2_ larger. At locations (3) and (4), the ratio *λ*_2_/*λ*_1_ becomes small.

**Figure 3:**
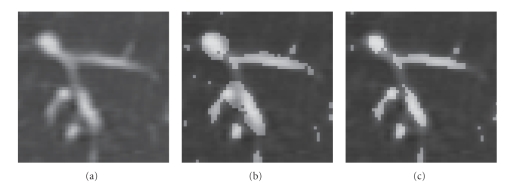
Segmentation results by thresholding the filter output with a fixed value (−600 [H.U.]). (a) Original CT image visualized using a window of 1500 H.U. at the level of −400 H.U. (b) A fixed $S=\{1,\sqrt{2},2,2\sqrt{2},4\}$ is used for all voxels. (c) (2) is used to determine **S**. In (b), nonvessel regions around a junction are also extracted as vessels whereas segmented regions reside within the visible vessels in (c). It should be noted that while vessel segments do not appear to be connected in this image, they are connected in the neighboring slices. In the event that they were not connected, the local disconnections would be fixed by the tracking process described in Section 2.2.

These criteria capture the characteristics of the model so as to differentiate tube structures from sheet and blob shaped structures when a point is within a vessel segment and close to its center. However, pulmonary vascular trees include many junctions, and criteria 2 and 3 above are not always satisfied around the branchpoints. *λ*_1_ and *λ*_2_ do not always satisfy criterion 3 above, especially in the thin segments and around the junctions. In addition, *λ*_3_ sometimes takes large positive values even when a point is within a straight segment, which implies criterion 2 is not always met. Figure 2(a) shows a trajectory by a white tube starting from a point demarcated by the left most white arrow. The tracking front advances left to right in the figure. The gray parts on the trajectory indicate the points where *λ*_3_ takes on negative values. These approximate to the junction locations. Figure 2(b) shows changes of the three eigenvalues of the Hessian matrix as the front point of the trajectory advances. *λ*_1_ and *λ*_2_ always take on large negative values compared to *λ*_3_. Intensity gradually changes towards junctions when the point is in a straight segment and it may cause *λ*_3_ to take on a positive value. When the point is around a junction, intensity decreases rapidly towards the segmental direction and it causes *λ*_3_ to take on a negative value. For these reasons, *λ*_3_ fluctuates along the trajectory. Also, *λ*_2_ oscillates with its phase opposite to *λ*_3_ since *λ*_2_ takes relatively smaller values when the point is around junctions. Based on this observation, the filter output function *F*(**x**) is defined as follows:

(1) $$\begin{array}{c} F\left(x\right)=\underset{\sigma_{f}\in S}{\max\;}-\frac{\sigma_{f}^{2}\lambda_{2}}{I\left(x\right)}, \end{array}$$

where *σ*_*f*_ is the standard deviation of a Gaussian function convoluted with an image so as to take second derivatives in the volume coordinate, and *I*(**x**) is the intensity value at the point. **S** is a discrete set of *σ*_*f*_ for multiscale integration. This equation only takes into account criterion 1. Thus, it may also enhance blob structures, typically image noise as well as cylindrical structures. According to our experience, in thoracic CT images, the noise will not be enhanced to the same degree as are vessels since contrast between parenchymal background and pulmonary vessels is relatively high compared to the noise. Most of the visible thin vessel segments exhibit intensity over −700 H.U. (Hounsfield Units) while lung parenchyma is typically between −800 and −900 H.U. This results in a 100 to 200 H.U. difference. Since Gaussian noise with standard deviation *σ* = 20 can be considered typical for CT images [8], a difference greater than 100 H.U. is not caused by noise. The noise can be eliminated by postprocessing discussed in Section 2.3. *F*(**x**) takes a maximum value when *σ*_*f*_ is the closest to the radius of a target segment among other *σ*_*f*_ in the range **S**. Therefore, **S** should include appropriate values that cover all the radii of the vessel segments in the lung. However, when **S** contains a wide range of values, and the filter output is calculated at a nonvessel point close to a junction or multiple thin segments are close to each other, *F*(**x**) gets larger than it should. This is caused by a Gaussian function with a large *σ*_*f*_ that excessively smoothes the region. Figure 3(b) shows the result when a fixed range of **S** was used for all voxels in the image. The filter overly extracted nonvessel regions around the junction. In order to avoid the inappropriate enhancement, large *σ*_*f*_ should be included in **S** only when it needs to be used for the detection of a thick segment. This requires a priori knowledge of the thick vessels. In thoracic images, thick vessels can easily be extracted by a simple intensity-based thresholding. We applied thresholding with a fixed value to obtain thick segments, and a discrete distance transform is applied to estimate an approximate radius *r*. By using a distance transform value *d*, the radius *r* is estimated as $r=\sqrt{d+1}-1$ [voxel]. We used −600 [H.U.] for both TLC and FRC scans as the threshold value. Using the radius information, range **S** is determined depending on the distance *d* as follows:

(2) $$\begin{array}{c} S=\{\begin{array}{cc} \left\{1\right\}, & 0<r\leq 1,\\ \left\{1,\sqrt{2},2\right\}, & 1<r\leq 2,\\ \left\{1,\sqrt{2},2,2\sqrt{2}\right\}, & 2<r\leq 2\sqrt{2},\\ \left\{1,\sqrt{2},2,2\sqrt{2},4\right\}, & 2\sqrt{2}<r. \end{array} \end{array}$$

Figure 3(c) shows the result using (2). The segmented region properly resides in the visible vessels. 

#### 2.1.2. Lung Segmentation

The filter output function takes large values not only at the tube structures but also at the edges of large concave structures such as bones and the heart. In addition, the function takes a relatively long time to be calculated for a whole volume. Typically, the lung occupies only 25% of the entire volume. Therefore, lung segmentation significantly reduces the computational cost of the tube enhancement filter. For simplicity, a lung segmentation method based on intensity thresholding and 3D labeling is employed. Obviously any existing lung segmentation algorithms such as [17] can also be used for this purpose. 

 The lung segmentation requires three seed points. They should be located in the left lung, right lung and trachea, respectively. Both left and right lungs are extracted by a simple thresholding with a fixed value and 3D labeling with the two seed points in the lung. The trachea and two or three subsequent branches are also segmented by thresholding with a fixed value and 2D labeling based upon connectivity between neighboring slices and the seed point. This airway segmentation is performed to eliminate the trachea from the lung segmentation result. Although the segmentation results from this very simple approach often contains part of the mediastinal region, it significantly reduces the number of voxels outside of the lung which would have otherwise been processed in the filtering step. It also greatly reduces the possibility of extracting false vessels caused by structures outside of the lung region. In our experiments, it took well under a minute to perform the lung segmentation while the lung segmentation served to reduce the tube enhancement filter processing time by more than four minutes.

### 2.2. Connectivity Improvements

Initial segmentation can be obtained by thresholding the filter output. However, since the segmentation works on a voxel-by-voxel basis, the result contains local disconnections and small holes. These are caused mainly by image noise and the difference of the intensity distribution on a cross-sectional plane between straight segments and junctions. The cross-sectional shape of a vessel contour at a junction becomes an ellipse. It causes lower filter output and local disconnections around junctions, especially where very thin segments bifurcate from a thick segment. The vessel traversal approach is suitable for improving connectivity and for filling small holes in a segment. The connectivity improvements include the following processes:

- initialization including automated localization of seed points,
- tracking terminations at junctions,
- radius estimation for boundary recovery.

#### 2.2.1. Initialization

The vessel traversal requires a set of seed (starting) points. Since points near the center of a segment are generally less influenced by noise, seed points are preferentially identified at segment centers. According to the vessel model, the intensity function takes a local maximum at the center of a segment. The local maximum position **x**_max _ in the volume coordinate can then be calculated by the following equation [18]:

(3) $$\begin{array}{c} x_{\max\;}=x+p=x-\frac{\left(\nabla I,e_{1}\right)}{\lambda_{1}}e_{1}-\frac{\left(\nabla I,e_{2}\right)}{\lambda_{2}}e_{2}, \end{array}$$

where ∇*I* is a gradient vector at **x**, **e**_1_ and **e**_2_ are eigenvectors corresponding to the eigenvalues *λ*_1_ and *λ*_2_. The operator (, ) takes the inner product of the two vectors in the equation. ∇*I* is estimated by using *σ*_*f*_ which maximizes (1) in *S* (i.e., the gradient is calculated as a convolution of the first derivative of a 3D Gaussian function and *σ*_*f*_). Since estimation of **x**_max _ is sensitive to image noise, **x** is considered to be located close enough to the center when the following conditions are met:

(4) $$\begin{array}{c} p_{x}\leq 0.5,\;\;p_{y}\leq 0.5,\;\;p_{z}\leq 0.5, \end{array}$$

where *p*_*x*_, *p*_*y*_, and *p*_*z*_ are the components of the vector **p**. Nonetheless, many points in the airway wall are also included as tracking seed points if only these conditions are taken into account, because the local intensity structure on the airway wall is quite similar to that of a thin segment. Many of those points in the airway wall can be differentiated since the cross-sectional shape at the points is elliptical compared to that of the thin vessel segments. Therefore, an additional condition is applied

(5) $$\begin{array}{c} \frac{\lambda_{2}}{\lambda_{1}}\geq 0.5. \end{array}$$

Since (3) is based on the cylindrical vessel model, estimation of the local maximum position is inaccurate if a point is close to a junction. In that case, all three eigenvalues of the Hessian matrix take negative values. Therefore, one additional condition is added

(6) $$\begin{array}{c} 0\leq \lambda_{3}. \end{array}$$

If the eigenvalues satisfy conditions (4), (5) and (6), then the point is considered to be the center of a vessel segment and is registered as a seed point for the tracking process.

#### 2.2.2. Termination Criteria

Starting from a seed point, the front position of a trajectory advances in the continuous 3D space of the volume coordinate according to the estimated tangent direction [8] with the step size of 0.2 voxel. At the seed point, *λ*_3_ is a positive value since the seed point satisfies condition (6). As the front position advances, *λ*_3_ changes its sign when it approaches a junction. A sign change of *λ*_3_ along a trajectory is illustrated in Figure 2. *λ*_3_ takes a negative value around the branchpoints and becomes positive when the front is remote from the junctions. The primary purpose of this tracking is to fix local disconnections associated with junctions. Therefore, once the front reaches a branchpoint, tracking is no longer needed. In addition, there are many seed points available for tracking. Thus, once a segment is connected to its parent segment, tracking can be terminated. This will prevent performing the tracking multiple times for a segment. 

**Figure 4:**
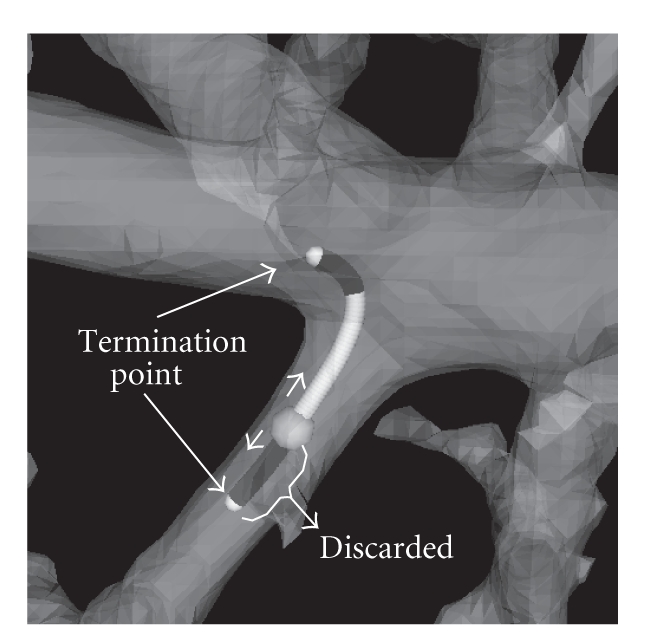
An illustration of the tracking process from a thin segment. The seed point is shown by a gray sphere. At the seed point, the tracking front can take both **e**_3_ or −**e**_3_ as the initial direction. Either way, tracking will be terminated after *λ*_3_ changes its sign twice and becomes a positive value. The sign of *λ*_3_ becomes negative before the front reaches a junction center. To ensure that the branch is reconnected by tracking, the process needs to wait until the sign changes twice—which indicates that the front truly passed through the junction area. Then the intensity value at the termination points is examined and either trajectory whose intensity value at the termination point is less than the other will be discarded. The other will be used for boundary recovery with the radius estimation.

 At a seed point, the tracking direction can be either **e**_3_ or −**e**_3_. One of them leads to a thicker segment and the other leads to a peripheral child branch. Local disconnection is often observed at a junction of a thick and a thin segment. Therefore, the tracking front should advance towards the thick segment in order to fill the potential gap between the two segments. Since a thick segment generally has higher CT values, the intensity value at the terminated point can be the criterion for selecting the trajectory which leads to the thicker segment. After tracking in both directions, the trajectory whose intensity value at the termination point is higher than the other is selected for radius estimation and the other is discarded. Figure 4 depicts the process.

#### 2.2.3. Radius Estimation

After tracking, spheres with an estimated radius are drawn at each tracking front position to fill possible holes and gaps between two segments. The intensity value around a point **x** can be estimated using the gradient ∇*I* and the Hessian matrix by the following quadratic equation;

(7) $$\begin{array}{c} I\left(x+\Delta x\right)\approx I\left(x\right)+\left(\nabla I,\Delta x\right)+\frac{1}{2}\left(\Delta x,H\Delta x\right). \end{array}$$

We determined Δ**x** as the radius of the sphere at the point when the estimated intensity value *I*(**x** + Δ**x**) reaches half value of *I*(**x**) from background value. Intensity decreases toward both the **e**_1_ and **e**_2_ directions and it tends to decrease faster along either **e**_1_ or −**e**_1_ compared with any other direction since the absolute value of *λ*_1_ is bigger than *λ*_2_. Therefore, Δ**x** is made parallel to **e**_1_ to estimate the smallest distance to the outside of the vessel from the point. Then, Δ**x** can be expressed as Δ**x** = *t* **e**_1_, where *t* is the minimum absolute value of the following equation:

(8) $$\begin{array}{c} t=\frac{-\left(\nabla I,e_{1}\right)\pm \sqrt{{\left(\nabla I,e_{1}\right)}^{2}-\lambda_{1}\left(I\left(x\right)-I_{\text{bg}}\right)}}{\lambda_{1}}. \end{array}$$

The background value is determined by *I*_bg_ = min *I*(**x** ± *iσ*_opt_ **e**_1_) where *i* = 1,2, 3 and *σ*_opt_ is the optimal *σ*_*f*_ which maximizes the filter output function (1) at the closest voxel from **x**. *σ*_opt_ at each voxel can be obtained in the vessel enhancement step and is reused to avoid expensive multiscale filtering in this step. The Hessian matrix and gradient vector are calculated by convoluting the second- and first- order derivative of Gaussian function with *σ*_opt_. The intensity value *I*(**x**) is obtained by convoluting Gaussian function with *σ*_opt_ at **x**, and *I*_bg_ is evaluated directly from the image using trilinear interpolation. The radius can be estimated at every front position. The minimum estimated radius along a trajectory is used for drawing spheres at each positions on the trajectory.

### 2.3. Connected Components with Many Branchpoints

The vascular tree can be characterized as an object with many branchpoints. An object that contains small numbers of branchpoints can be considered as noise or part of other structures. Therefore, a thinning algorithm [19] is applied to each object in the result to obtain the number of branchpoints in the object from its graph representation. Empirically determined, an object which has 100 or more branchpoints, is extracted as a vascular tree. The value 100 is chosen empirically by taking into account that the thinning algorithm may produce false branches.

## 3. Experiments

### 3.1. Materials

#### 3.1.1. Simulation

**Figure 5:**
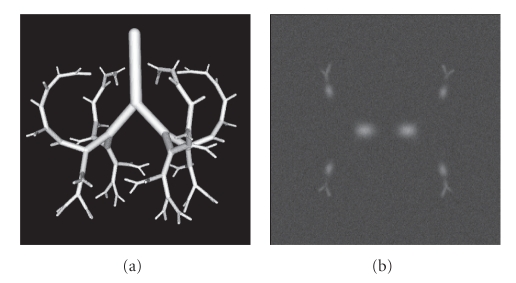
Visualization of the phantom data. (a) Surface display of the simulated vascular tree. (b) Cross-section of a phantom subjected to Gaussian noise of standard deviation *σ* = 30. The model contains 62 branchpoints and 125 segments. Intensity values at the center of the segments vary depending on the radius of the segment to mimic vessels in typical thoracic CT images. Gaussian noise with different standard deviations was added.

The algorithm was first applied to a set of computer-generated tree structures. Figure 5 shows the surface model of the tree structure and a cross-sectional image of one of the noisy phantom instances. This model was originally developed to represent an airway tree [20], yet it is appropriate also for use as a model for simulating a pulmonary vascular tree since the pulmonary arterial tree follows the airway tree out into the lung periphery and thus has the same general geometric relationships as the airway tree. The model contained 62 branchpoints and 125 segments. Each segment was characterized by a starting point, an end point and the associated radius. Branching angles were different for each bifurcation and their average was 83.9 degrees. The segments become thinner every time they bifurcate. 3D Gaussian spheres were moved along the linear segments to generate the tree structure in a 3D image whose dimension was 250 × 250 × 250 voxels. The radius of a segment decreases from the starting point to the end point of a segment so that it becomes the same as the radius of a child segment in order to allow smooth connection. Standard deviation of the 3D Gaussian spheres *σ*_*r*_ changes as the radius varies. In this phantom setting, maximum *σ*_*r*_ was six voxels and minimum *σ*_*r*_ was approximately one voxel in the volume. We also rotated the model in 11 different angles in 3D space and generated the structure in 3D images. Intensity value at the center of a segment mildly decreased from the starting point of a segment toward its end point along the tangent direction. This made thick segments brighter compared to the thin segments. The thickest segment had a value of −200 [H.U.] at the center whereas the thinnest had a value of −700 [H.U.] at the center. The background value was −900 [H.U.] to simulate a typical background intensity in the parenchymal region in lung CT images. Gaussian noise with a standard deviation *σ* = 20, 30, 40 was added to each image. After the result was obtained, a thinning method [19] was applied to obtain its graph representation so as to evaluate how many branches were correctly extracted and how many false branches were extracted by the segmentation algorithm. Both missing and extra branches were counted manually. 

#### 3.1.2. Clinical Data

The segmentation algorithm was applied to 44 CT scans from a total of 22 human subjects with lung volume held at 85% (for simplicity referred to as total lung capacity or TLC) and 20% (referred to functional residual capacity or FRC) of the subject's vital capacity. Both TLC and FRC scans were performed without imposition of an X-ray contrast agent. Subjects were healthy volunteers except for three who had mild chronic obstructive pulmonary disease (COPD) judged both by pulmonary function tests and visual assessment of the CT images. All images were scanned by a 4-slice MDCT scanner (Philips Mx8000, Philips Medical Systems, Cleveland, Ohio). In-plane pixel sizes of the CT images ranged from 0.52 mm to 0.88 mm, slice thickness was 1.3 mm, and slice increment was 0.65 mm. Trilinear interpolation was applied to obtain isotropic voxels. The resulting voxel dimension after the interpolation was about 0.6 mm. Approximately 550 and 500 slices per case were available for each TLC and FRC scan, respectively. 

**Figure 6:**
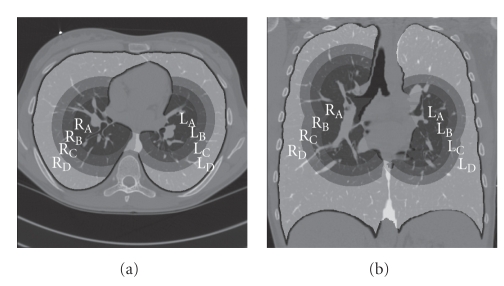
Eight distinct regions were delineated based upon their radial distance from the hilum. Two points were manually placed in each lung as landmarks denoting the hilum and the lung was three-dimensionally divided into a total of eight regions depending on the distance from the hilar points. Note that this interaction step is only used for the validation purposes.

 In order to evaluate the segmentation results quantitatively, more than 1000 points in the vessels were manually identified in each CT data set to form a validation set to assess the true positive (TP) rate. The points were defined by an experienced observer trained and supervised by a pulmonologist. TP rate is defined as the ratio between the total number of points detected by the algorithm and the total number of points in the data set. We consistently chose the center point of the vessels as a member of the TP point set because the center line is more important than vessel borders in defining the vessel geometry. Each lung was divided into four distinct regions in terms of radial distance from the hilum so as to avoid biased distribution of the test points. At least 100 points were identified in each region. Figure 6 shows the four regions in each lung. Region A is the closest to the hilum and region D is close to the pleural surface. Region A contains a greater number of thicker segments than the other regions. 

 To evaluate the false positive (FP) rate, 150 points were randomly placed in each region, and the points within the vessels were manually eliminated such that all points represented nonvessel regions. This left approximately 120 points per region which were located in the parenchyma, airway walls and pulmonary fissures. False positive rate is defined as the ratio between the total number of points included by the segmentation result and the total number of points in the data set.

### 3.2. Implementation Issues

We implemented this algorithm with a multithreaded process for the tube enhancement filter and the vessel traversal. Since the filter works on a voxel-by-voxel basis, it can be processed in parallel. Similarly, the vessel traversal from a seed point can be executed in parallel without depending on other seed point locations. Other processes such as lung segmentation and connected component analysis were implemented as serial processes. We used a Linux-based computer with dual Xeon 3.6 GHz processors (hyperthreading on) and 4 GB of memory for our experiment. The processing time varies depending on the size of the lung to be processed. Therefore, TLC scans take longer time than processing FRC scans. It took less than one minute (30 to 50 seconds) to obtain lung segmentation result. Then it took about one to three minutes to complete the tube enhancement filter process. The vessel traversal took 15 to 20 minutes. The connectivity components extraction calculations were on the order of one minute. The overall process required between 20 to 25 minutes to obtain a final result. The memory requirement is on the order of three times that required to hold the original volume. In our implementation, the program required approximately 900 MB for processing a volume consisting of 512 × 512 × 595 voxels.

### 3.3. Results

#### 3.3.1. Simulation

**Figure 7:**
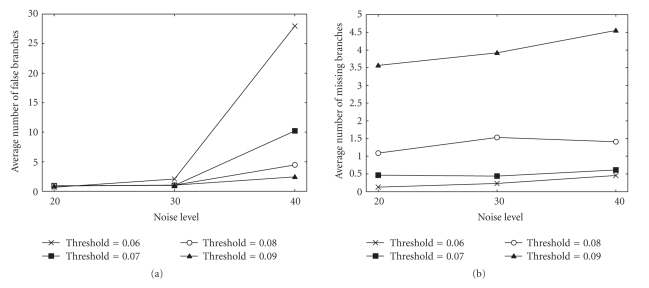
Average number of false (extra) branches and missing branches of the phantom evaluated by graph representation of the segmentation results. The false branches and missing branches were counted manually. (a) A total number of false branches (false positives). (b) A total number of missing branches (true negatives).

The segmentation algorithm has one major parameter, the threshold of the filter output used to obtain the initial segmentation. When it is decreased, more vessels and noise elements are extracted, increasing the TP rate as well as the FP rate. On the contrary, less vessels (and noise elements) are extracted if the threshold is increased, causing a lowering of the TP and FP rates. Since the threshold is a tradeoff between TP and FP rates, four thresholds were selected empirically and were tested by use of the simulation. Figure 7 shows average number of false (extra) branches and missing branches as a function of the threshold value. When the threshold setting was 0.06, the segmentation results were missing less than one branch per volume on average. However, the number of extra branches increased rapidly when noise levels went up. On the contrary, when the threshold was 0.09, the results missed 3.5 to 4.5 out of 125 branches on average whereas they had less extra branches than other threshold settings. In [8], a noise level *σ* = 20 was used to represent typical noise in CT images. For comparison, *σ* = 40 represents noise in ultrasound images. When the threshold was 0.07, the algorithm missed less than one branch on average for all noise levels and the results contained one or less extra branches in the case of *σ* = 20, 30. Derived from this result, a threshold value of 0.07 was used for the following experiments using the clinical data sets. 

#### 3.3.2. Clinical Data

**Figure 8:**
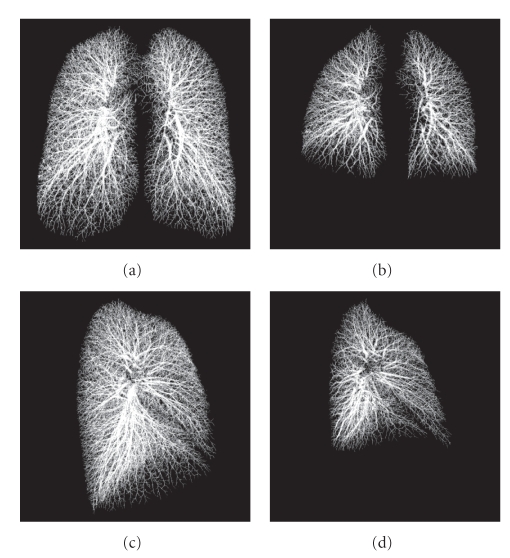
Two examples of segmentation results. (a) and (c) are the results from a TLC scan. (b) and (d) are from the associated FRC scan of the same subject. All images are shown at the same scale. Note the breathing-related changes of lung vasculature caused by regional lung parenchymal expansion between the TLC and FRC lung volumes.

Figure 8 shows volume-rendered images of the segmentation results from both TLC and FRC scans of one subject. Both results include peripheral thin segments close to the pleural surface. Since the scale of these images is the same, they also show how the lung geometry changes between the two volumes scanned. Table 1 shows a summary of the TP rate for both TLC and FRC scans. TP rate for TLC scans was 99.6% from the total of 16933 validation points and 99.5% from 15,281 points in right and left lungs, respectively. Similarly, TP rate for FRC scans was 99.0% from 13356 points and 98.2% from 10646 points in right and left lungs, respectively. Since the lung volume of FRC scans is less than that of TLC scans, there were less validation points for FRC data sets. Also, especially in the FRC scans due to increased compliance, motion artifacts may have contributed to a small increase in the error rate. Table 2 shows a summary of the FP rates for both TLC and FRC scans. A total of 11,925 points and 9620 points were used in right and left lung of the TLC scans, respectively, yielding FP rates of 1.21% in the right and 0.99% in the left lung. For FRC scans, a total of 11752 points in right lung and 8722 points in left lung were used and FP rates of 0.96% and 0.88% in the right and left lungs were obtained.

## 4. Discussion

In this section, the parameters affecting segmentation results will be discussed first followed by the performance difference between the left and right lungs. We will also discuss the difference of the validation results between TLC and FRC scans. Finally, we summarize the new contribution of the reported method.

### 4.1. The Threshold for Obtaining Initial Segmentation Result

The threshold for obtaining initial segmentation from the filter output influences the final result—when set close to zero, more objects including noise and other structures are obtained; less vessel segments and less noise result for larger threshold values. The threshold value thus affests the mehod's sensitivity and specificity. In the simulation, four different values were used to determine an appropriate value for pulmonary vascular segmentation from clinical CT images. 0.07 was empirically determined to be the best choice and was used for in vivo human images; 0.08 was shown to be an alternative based on the results of the simulation. When 0.08 is used for in vivo human lung images, the TP rate was 99.3% and 97.0% for TLC and FRC scans, respectively. The FP rate was 0.76% and 0.50% for TLC and FRC scans, respectively. In TLC images, the TP rate decreased merely 0.3% whereas the FP rate dropped by 0.45%. Therefore, the 0.08 setting was particularly appropriate for TLC images.

### 4.2. The Difference in TP and FP Rates between the Left and the Right Lungs

The TP rate in the region A for both TLC and FRC scans was lower than in the other regions. Region A is close to the hilum and contains many thick vessels. This region also contains thin segments bifurcating from the thick segments, some of which were missed by the segmentation. Radii of those missing segments were typically 1 to 2 mm and they emanated from segments whose radii were 5 to 8 mm. Vessel traversal was sometimes terminated prematurely before the front merged to the centerline of the thick segments. This observation calls for an additional traversal improvement in such highly asymmetric branches. In addition, some data sets had cardiogenic motion artifacts, particularly in the left lung regions, which caused branches to be missed. 

 The FP rate in region A was noticeably higher than in the other regions. This was mainly caused by thick airway walls in the region. Anatomically, many arterial segments run along with the airway segments, and sometimes they appear attached to each other in the CT image. This causes misdetection of the airway wall. Complete elimination of the airway wall may require a priori knowledge of the location of the airway tree. An integration of the airway tree segmentation [21, 22] will likely solve this problem.

### 4.3. The Difference in TP and FP Rates between TLC and FRC Scans

Image quality between TLC and FRC scans is substantially different. The lung is less dense at TLC than FRC and parenchymal density at TLC is more homogeneous. In FRC scans, parenchymal density increases and becomes less homogeneous. The density of the parenchyma increases typically by 100 to 200 Hounsfield units compared to that in the TLC scans. The image quality of FRC scans is also degraded by motion artifacts caused largely by cardiogenic motion which is accentuated by the fact that the lung is more compliant. The motion artifacts are more prominent in the left lung both because the heart is usually leftward shifted within the thorax. The overall TP rate in the FRC scans was less than that in the TLC scans by 0.6%.

### 4.4. Applications

The pulmonary arterial tree feeds the pulmonary capillaries which in turn drain into the pulmonary veins. The segmentation results demonstrated here contain only one or two connected trees in each lung, inferring that they may be implicitly connected in some locations. The total number of generations in the segmented trees can serve as a clinically important index. However, the arterial and venous trees must be evaluated separately. It is difficult to automatically estimate the number of generations in the segmented trees because of the implicit connections. This calls for an algorithm that separates arterial and venous tree. 

 The visual evaluation of the segmentation results indicates inclusion of peripheral thin segments close to the pleural surface. The algorithm, as currently completed and described, can be useful for clinical applications which do not require arterial and venous separation such as the detection of pulmonary emboli [23], the evaluation of the pulmonary vasculature in pulmonary hypertension and in the characterization of pulmonary nodules [24]. 

**Figure 9:**
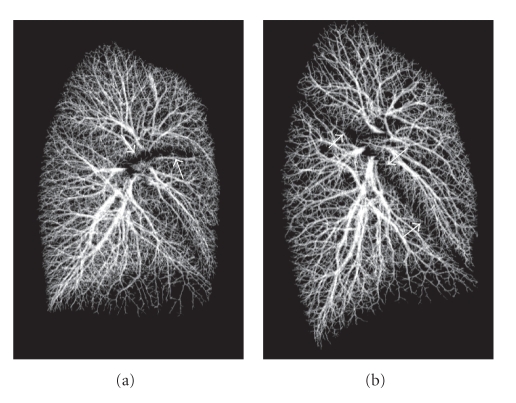
The extracted vascular tree of the right lung. Horizontal fissure is recognizable by the sparse region of the vascular tree in (a). Similarly, void region of the vascular tree describes the existence of oblique fissure in (b).

 An interesting byproduct of the vascular tree segmentation is that it depicts the pulmonary fissures as regions void of the vessel segments. Pulmonary fissures are visible structures in the lung, which separate each lung into lobes. The left lung has two lobes and the right lung has three lobes. Pulmonary fissures are very thin spaces and are often obscured by partial volume effects. Figure 9 shows the fissures visible in the segmentation results as void regions. Lobe segmentation has required fissure detection based upon identification of the fissure itself as has been reported in [25, 26]. Rough localization of pulmonary fissures using the vascular tree segmentation results avoids detection of the fissure itself and can be performed by searching in the sparse region of the vessel segments. This helps in limiting the region of interest to search for exact pulmonary fissure locations and may help to provide a fissure definition in the cases where the actual fissure is incomplete. 

 The main purpose of the presented paper was to report on a practical method for extraction of pulmonary vessels together with its validation on clinical CT images. The main novel aspects of the reported work are the following.

- Development of a simple yet practical tube enhancement filter output function (1).
- Seed point determination for vessel traversal from output of the tube enhancement filter.
- Fast radius estimation based on the quadratic Taylor expansion.

## 5. Conclusion

We have developed an algorithm to extract the pulmonary vascular trees from thoracic 3D CT images. The algorithm was applied to 44 volumetric CT scans consisting of 22 high volume scans and 22 low volume scans. It yielded 99.6% TP rate and 1.2% FP rate for the high volume scans, 98.6% TP rate and 0.9% FP rate for the low volume scans. The values for the TP and FP evaluations of these in vivo human data sets show a reliable performance and suitability of the proposed algorithm for future clinically relevant applications.

## Figures and Tables

**Table 1:** True positive rate in TLC and FRC scans.

	Left					Right					
	A	B	C	D	Total	A	B	C	D	Total	Overall

TLC	98.8%	99.8%	99.6%	99.7%	99.5%	99.4%	99.8%	99.7%	99.5%	99.6%	99.6%
FRC	97.8%	98.5%	98.1%	97.8%	98.2%	99.0%	99.3%	99.2%	98.6%	99.0%	98.6%

**Table 2:** False positive rate in TLC and FRC scans.

	Left					Right					
	A	B	C	D	Total	A	B	C	D	Total	Overall

TLC	1.48%	1.00%	0.73%	0.86%	0.99%	2.09%	1.08%	1.39%	0.99%	1.39%	1.21%
FRC	1.47%	0.86%	0.53%	0.86%	0.88%	1.37%	0.95%	0.73%	0.77%	0.96%	0.93%
